# Analysis for health system resilience against the economic crisis: a best-fit framework synthesis

**DOI:** 10.1186/s12961-025-01285-0

**Published:** 2025-03-14

**Authors:** Zeynab Foroughi, Parvin Ebrahimi, Shahram Yazdani, Aidin Aryankhesal, Majid Heydari, Mohammadreza Maleki

**Affiliations:** 1 Education Development Center, Iran University of Medical Sciences, Tehran, Iran; 2 School of Health Management and Information Sciences, Iran University of Medical Sciences, Tehran, Iran; 3 Virtual School of Medical Education and Management, Shahid Beheshti University of Medical Sciences, Tehran, Iran; 4 School of Health Sciences, Faculty of Medicine and Health Sciences, University of East Anglia, Norwich, UK; 5 National Agency for Strategic Research in Medical Sciences Education, Ministry of Health and Medical Education, Tehran, Iran

**Keywords:** Health system, Resilience, Economic crises

## Abstract

**Introduction:**

Countries, especially developing countries, are prone to economic crises, which are the consequences of various crises, including pandemics, climate crises, armed conflicts and migration. Therefore, policy-makers need a guiding framework for policy-making against the economic crisis that contributes to health system resilience. This study aimed to provide a holistic framework that guides health system policies before or during an economic crisis.

**Method:**

The study utilized the best-fit framework synthesis to enhance and adapt the Resilience Analysis Meta-Framework (RAMF) in the context of an economic crisis. The study analysed and compared the experiences of three high-income countries and three low-middle-income countries with the greatest diversity in terms of their context, shocks that caused the economic crises and their responses to them. The framework was expanded and adjusted on the basis of the adopted policies in the context of the economic crisis.

**Results:**

The adapted RAMF provides a holistic framework which shows the priority and relationships of various policy alternatives in each health system building block. This framework can be used as a guide to analyse any policy solution against the economic crisis by considering its necessary antecedent policies and consequence policies in other health system building blocks.

**Conclusions:**

Awareness in a health system via adapting appropriate cost control policies and governance structure can contribute to evidence-based cost control in all health system building blocks and need-based financing, drug and medical equipment procurement, human resource planning and service provision.

## Introduction

Health systems experiencing multiple crises have given rise to various definitions of health system resilience [1–4]. In this context, Hollnagel et al. [1] introduced the “four cornerstones” framework as part of theories of health system quality, while Blanchet et al. [2] provided a framework for defining the health system resilience capacities. Barasa et al. [3] introduced the concept of “everyday resilience”, and Kruk et al. [4] proposed a framework for measuring a “resilience index”. Subsequently, numerous studies have addressed the conceptual dispersion and the need for clearer frameworks and definitions of health system resilience [5–10]. Consequently, many researchers have sought to create a comprehensive framework for assessing, measuring and studying the resilience of health systems [11–14]. However, it is currently emphasized that health system resilience should be adapted to related contexts and specific crises to achieve a common understanding and cross-comparisons [15–18]. Therefore, theories of health system resilience should be applied to a wide range of crises, considering their specific characteristics into account [9].

Recently, the economic crisis caused by the pandemic, migration, armed conflicts and climate crisis has significantly impacted health systems, particularly in low-income countries [17, 19, 20]. The economic crisis refers to the disparity between certain economic indicators and a predetermined threshold such as GDP, liquidity, unemployment rate and currency value [21].

The experience of the economic crisis in various countries has revealed its effects in the reducing of health system revenue, diminishing of health insurance support and limiting of access to services. Simultaneously, economic crises increase health system costs, out-of-pocket payments, treatment concealment [22–26] and the number of end-stage, complicated and expensive hospital cases [27–30]. Therefore, there is a pressing need for a policy-making framework to build health system resilience in the face of economic crisis.

In this study, we analysed the health system resilience analysis meta-framework (RAMF) for its relevance in the context of an economic crisis [31]. The ultimate goal is to establish a more realistic framework for analysing and formulating policies regarding health system resilience against the economic crisis**.**

## Materials and methods

### Study design

This study applied a systematic literature review and best-fit framework synthesis method. This analysis is based solely on a literature review, and no data were collected in an anterograde manner. We applied the following steps regarding the best-fit framework synthesis method: First, we identified and defined the themes and codes of the health system resilience analysis meta-framework. Second, the relevant studies from selected countries were reviewed, and the health policies implemented to combat the economic crisis were extracted. In the third step, the extracted data were coded according to the a priori framework (that is, RAMF) [31–33]. Finally, the results that did not match the themes of the RAMF were analysed thematically.

### The a priori framework

There are various published health system resilience frameworks, and each one focuses on one or two dimensions of health system resilience, which jeopardizes its operationalization [31, 34]. The health system resilience analysis meta-framework (RAMF) is a synthetic framework for health system resilience analysis [35] that combines the core elements of various health system resilience theories, frameworks and models into one comprehensive framework, centred on the Six Building Blocks framework. However, further testing and field learning are still needed for its specific use [34, 36]. This meta-framework employs the ethnographic synthesis method, providing the opportunity to consider various published theories, frameworks and models within a new interpretive framework [31].

Also, in addition to introducing main themes to describe and analyse health system resilience, this framework indicated relationships among the phases and illustrated a dynamic framework [37].

The health system resilience analysis meta-framework (RAMF) comprises six primary dimensions. Table 1 provides definitions for the various components within these dimensions [31].

**Table 1:** The a priori framework dimensions and related codes [31]

Row number	RAMF dimensions	Codes	Definitions
1	Health system building blocks	Governance and leadership	Relevant policies pertain to policy formulation and decision-making mechanisms, stakeholder participation and preparation, responsiveness, regulation and accountability.
		Financing	Policies related to revenue collection, pooling, purchasing and allocation.
		Drug and medical equipment	Policies regarding the prioritization, selection, procurement, administration, consumption and distribution of drugs and medical equipment.
		Human resources	Policies pertaining to human resource planning, training and development and performance.
		Service delivery	Policies for determining the type, mix and level of service delivery, as well as the selection of health service providers.
		Information system	We considered it within other dimensions of health system resilience.
2	Resilience phases	Anticipation	The creation of arrangements, infrastructures or measures to identify and analyse risks and related system vulnerabilities through various methods.
		Preparation	The planning and development of various response plans and exercise scenarios, as well as the definition of leadership and command structures, and legal infrastructures to prepare for emergency situations.
		Response	Reactions of the health system to conditions induced by the crisis.
		Recovery	Policies that guide the health system in returning to its normal functions.
		Growth	Policies that contribute to sustainable change through learning, ensuring that future crisis do not cause damage to the system.
3	Resilience attributes	Awareness	The creation of arrangements and infrastructure to detect, monitor, interpret and disseminate information among various stakeholders.
		Surge capacity	Enhancing the capacity of health system building blocks to withstand the economic crisis, thereby ensuring the continuity of health system functions.
		Flexibility	Provision of alternative solutions and resources to respond to the effects of the crisis
		Resistance	Policies that maintain the stability of various health system functions without inducing change or improvement.
		Access to resources	Policies concerning the timely mobilization, acquisition and distribution of essential resources during a crisis.
4	Resilience tools	Risk analysis	Various retrospective and prospective methods for risk identification, analysis and assessment.
		Planning	Policies addressing with the development of contingency plans, regulations, roles and responsibilities, as well as the structure and functions of the health system.
		Monitoring	Policies aimed at establishing monitoring arrangements including methods for identifying and tracking crisis signals.
		Information and communication systems	Policies addressing the communicating, monitoring and control of various stakeholders through the design and implementation of relevant systems.
		Learning	Policies encompass various feedback mechanisms as well as methods for practice and experience.
		Institutionalization	Policies that employ new legal institutions or structured teams to address the crisis.
5	Resilience strategies	Absorptive strategies	Short-term strategies that maintain current health system methods and structures to preserve and restore its building blocks and functions.
		Adaptive strategies	Mid-term strategies that facilitate gradual changes across various health system building blocks.
		Transformative strategies	Strategies that enact long-term, significant changes in the structure and functions of health system.
6	Goals	Universal health coverage	Achieving universal health coverage is considered a primary goal of a resilient health system.

### Country selection and eligibility criteria

To achieve a comprehensive framework, we sought to include countries with the greatest diversity in terms of their context, the shocks that caused the economic crises and their responses to these crises. Therefore, the study was conducted in high-income European countries with three different financing systems (England, Spain and Germany) and three low- and middle-income Latin American countries (Brazil, Argentina and Cuba). Table 2 provides an abstract comparison of contextual factors among the selected countries.

**Table 2:** Comparison of selected countries’ contexts

Country name	Shock that caused economic crisis	Manifestations of economic crisis	Health service delivery system	Health financing system	References
Argentina	Significant economic fluctuations caused three periods of recession: in 1995, between 1999 and 2002, and since 2014.	• A significant increase in unemployment and poverty rates. • A decrease in the value of the current currency.	Integrated healthcare system	Consists of three main sectors: government, private and social security	[38–41]
Brazil	Economic turmoil and political instability in 2014.	• Decrease in GDP growth rate. • Increase in the population below the poverty line and the homeless. • Reduced access to private insurance.	Integrated healthcare system	Relying on private insurances	[42, 43]
Cuba	US sanctions against Cuba were initiated in 1961 and caused economic shock in 1980.	• Reduction in the import of oil as the main source of energy. • Decrease in gross domestic product. • Severe deficit of the government budget.	Relying on primary healthcare and polyclinics that provide comprehensive preventive services and primary care at the local level for individuals and families	Providing free services/socialist/financing on the basis of general taxes	[44–48]
England	The global economic crisis in 2008 led to a loan of 178 billion euros by the British government to prevent the collapse of the banking system. In 2010, the British public sector had a debt of 93 billion euros.	• Decreasing government per capita health spending. • Annual real expenditure growth of zero during the periods of 2009–2010 and 2014–2015.	National Health System	Providing free services/financing on the basis of general taxes	[49–51]
Germany	The global economic crisis in 2008 led to a projected deficit in the GKV health fund.	• Although the GDP per capita decreased in 2009, the country was not greatly affected by the economic shock. Health’s share of government spending from 2009 to 2011 was among the top five European countries.	Provides good access to services with freedom of choice of service provider and low waiting time, which is partly due to good infrastructure and a dense network of outpatient doctors and hospitals and a high level of quality of service provision	Most of its financing from government funds/compulsory social health insurance and participation of the labour market	[52–54]
Spain	The impact of the global financial crisis on this country began in 2008.	• Decreasing GDP growth rate and increasing unemployment rate from 2009 to 2013. • Among the countries of the European Union, it experienced one of the most severe crises and the worst consequences.	A healthcare system that provides a set of healthcare services from the central government and autonomous communities. In this system, responsibility of autonomous communities to plan and manage healthcare centres and service networks	Transforming the health financing system from social security (social insurance system) into a health system with financing based on general taxes	[24, 55–59]

GKV, Gesetzliche Krankenversicherung

Various shocks have led to economic crises in the selected countries. In low- and middle-income countries, factors such as the embargo in Cuba, political instability in Brazil, and economic fluctuations in Argentina have contributed to economic crises. In contrast, for the high-income countries – Spain, England and Germany – the global economic crisis was primarily triggered by the economic recession that began in 2007.

In terms of income levels and health indicators, the selected countries demonstrated varied responses to the crisis, largely due to differences in their health system financing structures.

The studies included an examination of the economic crisis in Spain, England and Germany following the global economic crisis of 2007, as well as in Cuba, Brazil and Argentina. Studies written in languages other than English, those that did not specify the policies of countries in addressing the economic crisis or those that outlined the effects of the economic crisis on health or disease were excluded.

### Search strategy

The search strategy included synonyms for “economic crisis”, “health system” and the names of the countries. The search was conducted in databases to identify studies published up until October 2023. Databases such as Scopus, Web of Science, Embase and PubMed were searched. Gray literature was identified using Google Scholar, Google search engines and the ProQuest database. The search strategy and results for each database are presented in Table 3.

**Table 3:** Search strategy and the results of databases

Database	Search strategy	Results
Web of science	TS = (“economic shock” OR “economic recession” OR recession OR “economic crisis” OR “financial crisis” OR “fiscal crisis” OR “banking crisis” OR “economic depression” OR “economic hardship” OR “economic insecurity” OR austerity OR “financial constraint” OR “economic downturn” OR “economic change” OR “economic breakdown” OR “economic turmoil” OR “economic stagnation” OR “economic adversity” OR “economic turbulence” OR “macroeconomic fluctuation” OR “economic crises” OR “financial crises” OR “budget scarcity” OR “restricted budget”) AND TS = (England OR UK OR "united kingdom" OR Spain OR Germany OR Cuba OR Argentina OR Brazil) AND TS = ("health system" OR "health care" OR healthcare)	464
PubMed	("economic shock"[Title/Abstract] OR "economic recession"[Title/Abstract] OR recession[Title/Abstract] OR "economic crisis"[Title/Abstract] OR "financial crisis"[Title/Abstract] OR "fiscal crisis"[Title/Abstract] OR "banking crisis"[Title/Abstract] OR "economic depression" [Title/Abstract] OR "economic hardship"[Title/Abstract] OR "economic insecurity"[Title/Abstract] OR austerity[Title/Abstract] OR "financial constraint"[Title/Abstract] OR "economic downturn"[Title/Abstract] OR "economic change"[Title/Abstract] OR "economic breakdown"[Title/Abstract] OR "economic turmoil"[Title/Abstract] OR "economic stagnation"[Title/Abstract] OR "economic adversity"[Title/Abstract] OR "economic turbulence"[Title/Abstract] OR "macroeconomic fluctuation"[Title/Abstract] OR "economic crises"[Title/Abstract] OR "financial crises"[Title/Abstract] OR "budget scarcity"[Title/Abstract] OR "restricted budget"[Title/Abstract]) AND ("health system"[Title/Abstract] OR "health care"[Title/Abstract] OR healthcare[Title/Abstract]) AND (England[Title/Abstract] OR UK[Title/Abstract] OR "united kingdom"[Title/Abstract] OR Spain[Title/Abstract] OR Germany[Title/Abstract] OR Cuba[Title/Abstract] OR Brazil[Title/Abstract] OR Argentina[Title/Abstract])	330
Scopus	TITLE-ABS-KEY ( "economic shock" OR "economic recession" OR recession OR "economic crisis" OR "financial crisis" OR "fiscal crisis" OR "banking crisis" OR "economic depression" OR "economic hardship" OR "economic insecurity" OR austerity OR "financial constraint" OR "economic downturn" OR "economic change" OR "economic breakdown" OR "economic turmoil" OR "economic stagnation" OR "economic adversity" OR "economic turbulence" OR "macroeconomic fluctuation" OR "economic crises" OR "financial crises" OR "budget scarcity" OR "restricted budget") AND TITLE-ABS-KEY ( england OR uk OR "united kingdom" OR spain OR germany OR cuba OR argentina OR brazil) AND TITLE-ABS-KEY ( "health system" OR "health care" OR healthcare)	1285
Econbiz	(title:("economic crisis" OR "economic crises" OR "financial crisis" OR "financial crises" OR recession OR depression AND title:"health system" OR "health care" OR "health sector" OR healthcare) AND title:("health system" OR healthcare OR "health care") AND title:(england OR uk OR "united kingdom" OR spain OR germany OR cuba OR argentina OR brazil))	386
ProQuest	abstract(“economic shock” OR “economic recession” OR recession OR “economic crisis” OR “financial crisis” OR “fiscal crisis” OR “banking crisis” OR “economic depression” OR “economic hardship” OR “economic insecurity” OR austerity OR “financial constraint” OR “economic downturn” OR “economic change” OR “economic breakdown” OR “economic turmoil” OR “economic stagnation” OR “economic adversity” OR “economic turbulence” OR “macroeconomic fluctuation” OR “economic crises” OR “financial crises” OR “budget scarcity” OR “restricted budget”) AND abstract("health system" OR "health care" OR "health sector" OR healthcare) AND abstract(England OR UK OR "united kingdom" OR Spain OR Germany OR Cuba OR Argentina OR Brazil)	103

### Selection process

EndNote software was utilized to organize the retrieved studies. After removing duplicates, two study authors conducted an initial screening based on the title and abstract, followed by a full-text screening based on the eligibility criteria.

### Data collection and analysis

The Mladovsky et al. framework was employed to identify policies implemented in response to the economic crisis. According to this framework, health systems adopt policies in three main areas to address the economic crisis: health costs, government participation in financing and the impact on health system goals [60]. The identified policies were analysed using deductive coding according to RAMF. This analysis was conducted by uploading studies into MAXQDA 2020 software. Consistency among researchers was ensured by using Table 1 to present study codes and their definitions. Results that did not match the a priori framework were coded inductively. Authors engaged in discussions about coding, and the coding process was conducted iteratively.

### Quality appraisal

The MAAT quality appraisal tool (version 2018) was utilized owing to the inclusion of studies with diverse qualitative and quantitative designs. Studies were appraised and scored on a five-point scale (0, 25, 50, 75 and 100) by two authors (ZF, MM) [61, 62]. Disagreements were resolved by the opinion of a third researcher (A.A.). No study was excluded owing to low quality, as this qualitative study considers any adopted policies mentioned to be valuable. However, greater weight was given to studies with higher quality ratings in our interpretations in cases of any contradictions.

## Results

### Included studies

**Fig. 1:**
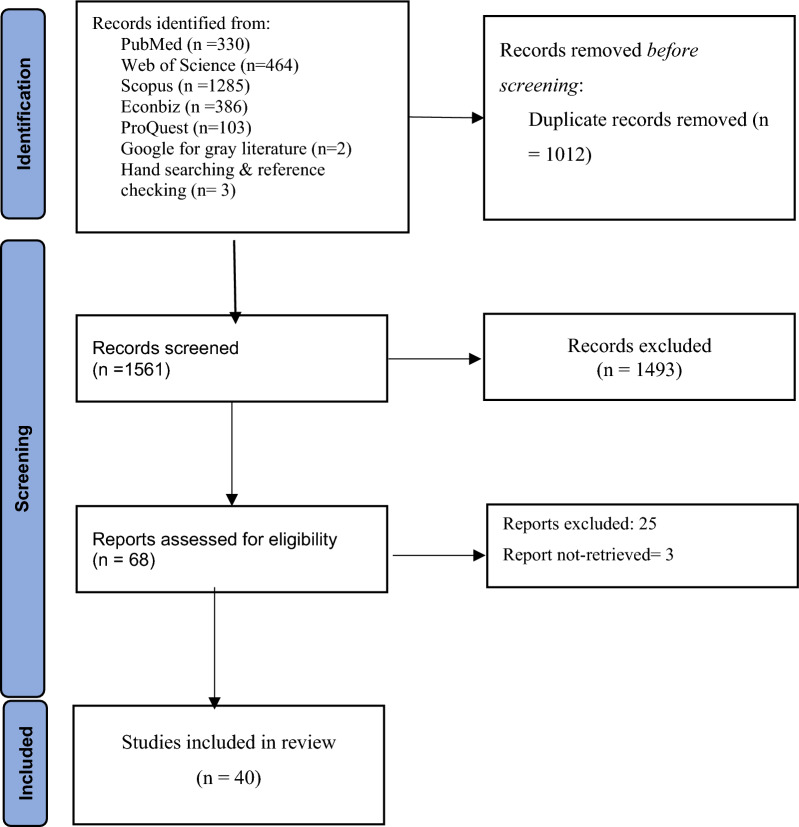
PRISMA 2020 flow diagram

Out of the initially detected 2573 studies, 40 were included for review (Fig. 1). The majority of these studies were conducted in Brazil (27%) and Spain (25%). Relevant information for Cuba and England was also found in 17% of the studies for each country. Only 10% of the studies were related to Germany.

Different countries responded to the economic crisis in two distinct ways: through a reform approach or an austerity approach. Argentina, Germany and Cuba adopted the reform approach, while Spain and Brazil implemented the austerity approach. However, England’s healthcare system has adopted a combination of both approaches. These approaches served as the foundation for comparative analysis of different countries.

### Quality appraisal results

The quality appraisal indicated that 55% of the included studies were non-empirical, 35% of the studies had a high rating (75 or 100) and only 10% were rated low (50 or 25; Table 4).

**Table 4:** Included studies and their characteristics

Study	Country	Study design	Quality score
[63]	Brazil	Non-empirical	–
[64]	Spain	Qualitative	100
[65]	Brazil	Non-empirical	–
[24]	Spain	Cross-sectional analytic study	75
[66]	England	Non-empirical	–
[67]	England	Non-empirical	–
[68]	Brazil	Qualitative description	50
[69]	Spain	Cross-sectional analytic study	100
[46]	Cuba	Qualitative description	100
[70]	Spain	Non-empirical	–
[71]	Spain	Non-empirical	–
[72]	Spain	Qualitative description	50
[73]	England, Germany and Spain	Non-empirical	–
[74]	Brazil	Cohort study	100
[75]	Argentina	Cross-sectional analytic study	100
[76]	Cuba	Non-empirical	–
[39]	Argentina and Brazil	Case-study	100
[77]	England and Spain	Case studies	50
[78]	Argentina	Non-empirical	–
[79]	Argentina	Case studies	75
[80]	Cuba	Non-empirical	–
[50]	England	Non-empirical	–
[81]	Spain	Cross-sectional analytic study	100
[82]	Brazil	Non-empirical	–
[83]	Cuba	Cohort study	50
[84]	Cuba	Case study	100
[85]	Spain	Non-empirical	–
[86]	Spain	Systematic review	–
[87]	Brazil	Case study	100
[42]	Brazil	Case study	100
[88]	Brazil	Non-empirical	–
[52]	Germany, England and Spain	Case study	100
[54]	Germany	Non-empirical	–
[59]	Spain	Non-empirical	–
[58]	Spain	Non-empirical	–
[89]	Cuba	Non-empirical	–
[90]	Brazil	Case study	100
[91]	Cuba	Non-empirical	–
[92]	Brazil	Non-empirical	–
[53]	England and Germany	Non-empirical	–

### Analysing detected health policies according to RAMF dimensions

**Fig. 2:**
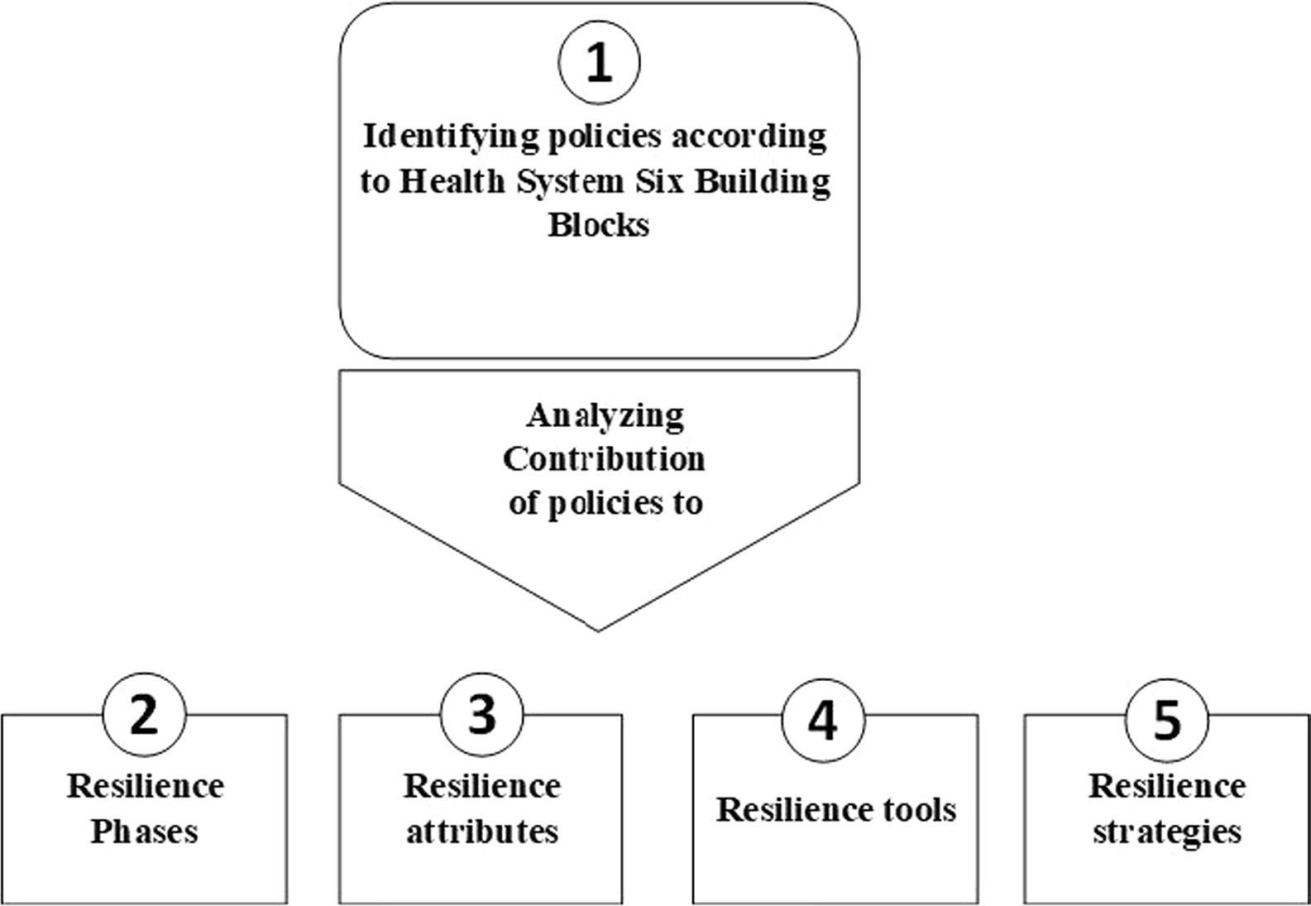
Policy analysis steps

The following presents the results of the analysis of selected countries’ health policies in response to the economic crisis (as seen in Table 1). As the health system’s Six Building Blocks framework is the core dimension of RAMF, policies have been identified and classified in each building block. Subsequently, their contributions to the other dimensions – resilience phases, attributes, tools and strategies – have been examined. Figure 2 presents the analysis steps.

## Policies adopted in each health system building block

### Governance policies

The governance policies addressing the economic crisis fall into three general areas: governance structure, the approach to public–private partnership and cost control policies.

#### Decision on governance structure

Some countries removed redundant structures (for example, Cuba and England) [77, 91], while others expanded less expensive ones (for example, Brazil and Spain) [57, 58, 93, 94]. Efforts were made to enhance coordination and integration among service delivery institutions [39, 54, 76, 89, 91, 95]. Additionally, decision-making in the health system varied, with some countries opposing centralization (England) and others supporting it (Argentina, Brazil and Spain) [39, 58, 95]. The approach towards governance structure determined the other two governance areas.

#### Decision on contribution of public and private sector

Countries exhibited divergent approaches to neoliberal policies. The countries with reform policies, such as Argentina and England, ceased their public–private partnerships (PPPs) [39, 50, 53, 66], while Cuba maintained its socialist policies and continued to offer tax-based services. Conversely, countries implementing austerity policies aimed to reduce government spending on healthcare and expand the utilization of PPPs (Spain and Brazil) [24, 93].

#### Cost control policies

Various cost control policies were adopted to manage the behaviour of the population, insurance organizations and providers in terms of resource consumption. In applying this approach, Cuba promoted the utilization of primary healthcare (PHC) [84], Brazil emphasized the private sector [93] and England and Spain focused on controlling hospital care costs [53, 59]. Table 5 illustrates the governance policies and their contributions to other health system resilience dimensions.

**Table 5:** Governance policies to counteract the economic crisis and their relationship to health system resilience components

Theme	Policies	Country	Resilience phases					Resilience attributes					
			Anticipation	Preparation	Response	Recovery	Growth	Awareness	Surge capacity	Flexibility	Resistance	Access to resources	Collaboration and coordination
Decision regarding centralization or decentralization	Recentralization of governance	Brazil [95]		*			*		*		*		*
	Centralization of control and management and removal of redundant structures	Argentina [39], Spain [58] and England [53]		*			*				*		*
	Restructuring ministries or other government institutions to reduce overhead and executive costs	England [77]		*			*				*	*	
	Decentralization of managerial responsibilities	England [53]		*			*						
	Integration of purchaser and provider	England [53]		*			*				*		*
Decision regarding PPP or privatization	Promoting privatization	Brazil [93] and Spain [24]		*			*		*	*	*	*	
	Stopping the use of market mechanisms and neoliberal policies	England [50] and Argentina [39]		*			*				*		
	Allowing foreign institutions to enter into joint ventures with Cuban government entities	Cuba [89]		*			*		*	*		*	*
	Promotion of private service utilization by the population through subsidization	Brazil [93]		*			*		*	*		*	

Theme	Policies	Country	Resilience tools										Resilience strategies		
			Risk analysis	Change in input/output level	Change in quality level	Legislation	Change in behaviour	Planning	Monitoring	Institutionalization	Learning	Information and communication systems	Absorptive	Adaptive	Transformative
Decision regarding centralization or decentralization	Recentralization of governance	Brazil [95]								*	*				*
	Centralization of control and management and removal of redundant structure	Argentina [39], Spain [58] and England [53]		*						*				*	
	Restructuring ministries or other government institutions to reduce overhead and executive costs	England [77]		*										*	
	Decentralization of managerial responsibilities	England [53]												*	
	Integration of purchaser and provider	England [53]				*			*				*		
Decision regarding PPP or privatization	Promoting privatization	Brazil [93] and Spain [24]		*						*			*		
	Stopping the use of market mechanisms and neoliberal policies	England [50] and Argentina [39]					*			*				*	
	Allowing foreign institutions to enter into joint ventures with Cuban government entities	Cuba [89]				*								*	
	Promotion of private service utilization by the population through subsidization	Brazil [93]					*						*		

### Financing policies

Financing policies were analysed in their three main functions: revenue collection, pooling, and purchasing or resource allocation. Decisions about revenue collection focused on contributions of households, the public sector, and/or the private sector. Regarding purchasing or resource allocation, policies were adopted to determine resources allocated to various health system levels or covered services.

#### Decisions regarding the contribution of households in revenue collection

Countries have adopted their approaches to revenue collection based on decisions regarding contribution of the public and private sectors and governance structures. Argentina and Germany have strengthened their health insurance systems, while Cuba and England continue to cover the healthcare service costs through general taxes [52, 54, 79, 96]. Austerity measures have led to reduced public insurance coverage and increased household contributions to financing in Spain and Argentina [24, 56, 59, 78, 79, 86].

#### Decisions regarding the share of government and private-sector contribution in health financing

Revenue collection policies are also adjusted on the basis of approaches towards the contribution of the public and private sectors. For example, the German government invested in insurance systems by empowering redistribution funds, which distribute contributions among various insurance funds, as well as in relation to drugs and medical equipment [52, 54, 96]. However, countries with an austerity approach decreased the share of GDP allocated to the health system and increased private-sector financing [75, 97].

#### Reducing spending on hospitals’ services

In line with cost control policies, these mechanisms are applied in the resource allocation function of health system financing. Specifically, Spain considered implementing a cost ceiling for pharmaceutical services [86], England reduced investment in hospitals [77], and Germany extended its Diagnosis Related Group (DRG) payment system to psychiatric hospitals [96].

#### Reducing benefit packages

Resource allocation is adjusted on the basis of needs assessments by establishing a relationship between insurance premiums and/or service franchises with income, or by considering specific benefits for vulnerable populations as seen in Brazil, Spain and Germany [42, 54, 59].

Table 6 presents financing policies and their contributions to other health system resilience dimensions.

**Table 6:** Financing policies to counteract economic crisis and their relationship to health system resilience components

Theme	Policies	Country	Resilience phases					Resilience attributes					
			Anticipation	Preparation	Response	Recovery	Growth	Awareness	Surge capacity	Flexibility	Resistance	Access to resources	Collaboration and coordination
Decision regarding the share of government and private-sector contribution in health financing	Public financing and free service coverage	Cuba [50]		*			*		*	*	*	*	
	Reducing the share of health system support from GDP and increasing private insurances	Brazil [93, 97]			*	*				*	*	*	
	Increasing households contributions	Brazil [97]			*	*				*	*	*	
	Increasing private-sector contribution	Argentina [75]			*	*				*	*	*	
Decision regarding the contribution of households to revenue collection	Linking copayments or the insurance premium to the income tithe	Spain [24, 56, 59]		*			*			*		*	
	Reducing the contribution of low-income vulnerable patients on insurance funds/	Germany [54]		*			*			*		*	
	Restructuring co-payment on the basis of services classification (basic, supplementary or accessory)/determining the franchise for supplementary and ancillary services	Spain [71, 86]		*			*			*		*	
	Introducing co-insurance for drugs	Spain [86]			*	*					*	*	
	Increasing the contribution rate of workers compared with employers to health insurance funds	Germany [52]			*	*				*	*	*	
	Making it mandatory to have private or government insurance	Germany [52]		*			*		*		*	*	
	Unification of contribution rates in all funds	Germany [54]		*			*			*		*	
Shifting pooling source	Increasing private insurance/developing private financing programs	Brazil [98] Spain [24]			*	*				*	*	*	
Empowering pooling source	Empowering the redistribution funds which distribute contributions among various insurance funds	Argentina [79], Germany [52, 54, 96]		*			*		*	*		*	*
	Improving insurance organizations through increasing their share by redistributing the insurance fund	Argentina [79]		*			*		*	*		*	*
Reducing the benefit package	Reducing resource allocation to governmental health promotion services	Brazil [97, 99]			*	*					*	*	
	Reducing the benefit package to more emergency services	Argentina [78, 79]			*	*				*	*	*	
	Providing benefits to people who are 65 years and older and disabled who are in the lower income deciles	Brazil [42]		*			*	*	*		*	*	
	More restricted eligibility criteria for free healthcare services	Spain [24, 56, 59]			*	*					*	*	
Reducing spending on hospitals and treatment services	Determining a health system spending ceiling	Brazil [42, 90, 98]			*	*					*	*	
	Determining a monthly cost ceiling for pharmaceutical services	Spain [86]			*	*					*	*	
	Reducing hospital costs through price reductions	England [77]			*	*					*	*	
	Reducing capital investment at hospitals	England [77]			*	*					*	*	
	Extending DRG	Germany [96]		*			*					*	

Theme	Policies	Country	Resilience tools										Resilience strategies		
			Risk analysis	Change in input/output level	Change in quality level	Legislation	Change in behaviour	Planning	Monitoring	Institutionalization	Learning	Information and communication systems	Absorptive	Adaptive	Transformative
Decision regarding the share of government and private-sector contribution in health financing	Public financing and free service coverage	Cuba [50]		*										*	
	Reducing the share of health system support from GDP and increasing private insurances	Brazil [93, 97]		*									*		
	Increasing households contributions	Brazil [97]		*									*		
	Increasing private-sector contribution	Argentina [75]		*									*		
Decision regarding the contribution of households to revenue collection	Linking copayments or the insurance premium to the income tithe	Spain [24, 56, 59]	*						*		*			*	
	Reducing the contribution of low-income vulnerable patients on insurance funds/	Germany [54]	*						*		*			*	
	Restructuring co-payment on the basis of services classification (basic, supplementary or accessory)/determining the franchise for supplementary and ancillary services	Spain [71, 86]	*						*		*			*	
	Introducing co-insurance for drugs	Spain [86]		*			*						*		
	Increasing the contribution rate of workers compared with employers to the health insurance funds	Germany [52]		*									*		
	Making it mandatory to have private or government insurance	Germany [52]		*		*	*						*		
	Unification of contribution rates in all funds	Germany [54]	*						*		*			*	
Shifting pooling source	Increasing private insurance/developing private financing programs	Brazil [98] Spain [24]		*									*		
Empowering pooling source	Empowering the redistribution funds which distribute contributions among various insurance funds	Argentina [79], Germany [52, 54, 96]		*									*		
	Improving insurance organizations through increasing their share by redistributing the insurance fund	Argentina [79]		*									*		
Reducing the benefit package	Reducing resource allocation to governmental health promotion services	Brazil [97, 99]		*									*		
	Reducing the benefit package to more emergency services	Argentina [78, 79]	*	*				*					*		
	Providing benefits to people who are 65 years and older and disabled who are in the lower income deciles	Brazil [42]	*												
	More restricted eligibility criteria for free healthcare services	Spain [24, 56, 59]				*	*						*		
Reducing spending on hospitals and treatment services	Determining a health system spending ceiling	Brazil [42, 90, 98]				*	*						*		
	Determining a monthly cost ceiling for pharmaceutical services	Spain [86]				*	*						*		
	Reducing hospital costs through price reductions	England [77]				*	*						*		
	Reducing capital investment at hospitals	England [77]				*	*						*		
	Extending DRG	Germany [96]	*			*					*	*		*	

### Drug and medical equipment

In response to the economic crisis, countries implemented policies to control the price and consumption of drugs and medical equipment; these were aimed at improving the access of vulnerable populations and ensuring production of essential items. Table 7 illustrates the policies related to drugs and medical equipment in response to the economic crisis and their contribution in other dimensions of health system resilience.

**Table 7:** Drug and medical equipment policies to counteract economic crisis and their relationship to health system resilience components

Theme	Policies	Country	Resilience phases					Resilience attributes					
			Anticipation	Preparation	Response	Recovery	Growth	Awareness	Surge capacity	Flexibility	Resistance	Access to resources	Collaboration and coordination
Promoting drug production	Government support in increasing Cuba’s capacity for continuous drug production	Cuba [89]		*			*		*	*		*	
	Increase in the price of imported drugs	Brazil [87]			*	*			*		*	*	
	Implementation of the MediCuba program to reduce the import of final pharmaceutical products and increase the import of raw chemicals	Cuba [89]		*			*		*	*		*	
Price control	Applying direct and indirect price reduction policies for drugs and medical equipment	England [77]			*	*					*	*	
	Reference pricing of drugs based on their cost–benefit assessment results	[96]			*	*					*	*	
	Promoting generic drug consumption	Argentina, England, Spain and Cuba [58, 71, 77, 78, 79]			*	*					*	*	
	Set of rules to reduce or control drug prices	Germany [52]			*	*					*	*	
Control of consumption	Introducing a 10% copayment for previously free outpatient drugs and increasing the patient’s share of payment for drugs	Spain [24, 56]			*	*					*	*	
	Introducing evidence-based prescription tools including clinical guidelines, electronic prescriptions, etc	England [77]		*			*		*	*	*	*	
	Reducing medical equipment purchasing	England [77] Spain [24]			*	*					*	*	
Improving access to essential medicine for vulnerable populations	Diversification of sources of supply of medicine and medical equipment	Cuba [46]			*	*			*	*	*	*	
	Improving access to medicines by subsidizing them since 2004	Brazil [87]			*	*				*	*	*	
	Implementing Remediar for providing essential drugs for vulnerable groups	Argentina [39, 75, 79]		*			*	*	*	*		*	
	Adding to the list of essential medicine	England [87]			*	*		*	*			*	
Evidence-based coverage	Improving evidence-based decision-making for coverage of cost–effective medicines	England and Brazil [63, 87]											
	Introducing HTA to determine drug and medical equipment coverage	England [77]		*			*	*					
	Creating standards for systematic evaluation of drug benefits/	Spain [52]	*			*	*						*

Theme	Policies	Country	Resilience tools										Resilience strategies		
			Risk analysis	Change in input/output level	Change in quality level	Legislation	Change in behaviour	Planning	Monitoring	Institutionalization	Learning	Information and communication systems	Absorptive	Adaptive	Transformative
Promoting drug production	Government support in increasing Cuba’s capacity for continuous drug production	Cuba [89]			*	*					*			*	
	Increase in the price of imported drugs	Brazil [87]		*		*	*							*	
	Implementation of the MediCuba program to reduce the import of final pharmaceutical products and increase the import of raw chemicals	Cuba [89]			*	*					*			*	
Price control	Applying direct and indirect price reduction policies for drugs and medical equipment	England [77]		*									*		
	Reference pricing of drugs based on their cost–benefit assessment results	[96]		*									*		
	Promoting generic drug consumption	Argentina, England, Spain and Cuba [58, 71, 77, 78, 79]				*	*						*		
	Set of rules to reduce or control drug prices	Germany [52]				*	*						*		
Control of consumption	Introducing a 10% copayment for previously free outpatient drugs and increasing patients’ share of payment for drugs	Spain [24, 56]		*		*	*						*		
	Introducing evidence-based prescription tools including clinical guidelines, electronic prescriptions, etc	England [77]			*	*	*		*			*		*	
	Reducing medical equipment purchasing	England [77] Spain [24]		*		*							*		
Improving access to essential medicine for vulnerable populations	Diversification of sources of supply of medicine and medical equipment	Cuba [46]		*									*		
	Improving access to medicines by subsidizing them since 2004	Brazil [87]		*									*		
	Implementing Remediar for providing essential drugs for vulnerable groups	Argentina [39, 75, 79]	*	*		*						*		*	
	Adding to the list of essential medicine	England [87]	*										*		
Evidence-based coverage	Improving evidence-based decision-making for coverage of cost–effective medicines	England and Brazil [63, 87]													
	Introducing HTA to determine drug and medical equipment coverage	England [77]	*	*		*		*	*	*	*			*	
	Creating standards for systematic evaluation of drug benefits/	Spain [52]			*		*	*	*	*			*		*

HTA, Health Technology Assessment

#### Price control

Cost reduction in this building block is achieved through pricing policies and evidence-based prioritization of drugs and medical equipment [96]. Additionally, countries have mandated the use of generic drugs as a cost control measure [58, 71, 77–79].

#### Control of consumption

Countries controlled the utilization of drugs and medical equipment by introducing co-payments, adjusting purchasing policies and providing treatment protocols and guidelines [24, 56, 77, 87, 89].

#### Improving access to essential medicine for vulnerable populations

Improving access to essential medicines for vulnerable populations was the aim of policies in almost all countries [39, 52, 75, 77, 79, 87, 96]. In Argentina and Brazil, access to essential medicines was secured for people, especially vulnerable groups, through subsidization [39, 75, 79, 87].

#### Promoting drug production

Through various policy initiatives, Cuba and Brazil controlled the consumption of imported drugs and incentivized the internal production of essential drugs [87, 89]. In fact, Cuba has developed its drug production capacity and research and development. This country focused on importing raw pharmaceutical materials instead of finished pharmaceutical products [89].

### Human resources

The majority of cost-reduction policies in the human resource area involved reducing the quantity [24, 59, 77, 93, 97, 100] and quality of human resources in Spain, England, Brazil and Germany by reducing payments, increasing the workload and imposing unfavourable terms of employment contracts [58, 59, 77, 96]. Conversely, Cuba and Argentina enhanced the quality and quantity of their primary care staff through education and training [39, 84, 89]. Table 8 presents human resource policies to counteract the economic crisis and their contribution in other dimensions of health system resilience.

**Table 8:** Human resource policies to counteract economic crisis and their relationship to health system resilience components

Theme	Policies	Country	Resilience phases					Resilience attributes					
			Anticipation	Preparation	Response	Recovery	Growth	Awareness	Surge capacity	Flexibility	Resistance	Access to resources	Collaboration and coordination
Reducing payments and benefits	Reduction of financial incentives and benefits/elimination of overtime/reduction of salaries and income control	Spain [58 and 59] England [77]			*	*					*	*	
	Reducing the bargaining power of employees and entering into agreements between employees	Brazil [97]			*	*					*	*	
Reducing human resources	Stopping the import of health professionals	Brazil [93, 97]			*	*					*	*	
	Reducing healthcare human resources	England, Spain [24, 77, 100]			*	*					*	*	
Reducing the cost of human resources	Increasing the workload/reducing in-service trainings/applying temporary employment contracts	England and Spain [24, 59, 71, 77]			*	*					*	*	
Increasing the quality and flexibility of human resources	Training new healthcare professionals as an alternative to their migration/exporting healthcare professionals and training foreign students	Cuba [84, 89, 91]		*			*		*			*	
	Increasing healthcare personnel’s flexibility to reduce costs/	Brazil [93, 97]		*			*		*	*		*	
	Training in health promotion and epidemiology concepts for hospital professionals	Cuba [91]		*			*		*	*			
	Training primary healthcare professionals	Argentina [39]		*			*		*	*			

Theme	Policies	Country	Resilience tools										Resilience strategies		
			Risk analysis	Change in input/output level	Change in quality level	Legislation	Change in behaviour	Planning	Monitoring	Institutionalization	Learning	Information and communication systems	Absorptive	Adaptive	Transformative
Reducing payments and benefits	Reduction of financial incentives and benefits/elimination of overtime/reduction of salaries and income control	Spain [58, 59] and England [77]		*	*	*							*		
	Reducing the bargaining power of employees and entering into agreements between employees	Brazil [97]		*	∞	*							*		
Reducing human resources	Stopping the import of health professionals	Brazil [93, 97]		*	*	*							*		
	Reducing healthcare human resources	England and Spain [24, 77, 100]		*	*	*							*		
Reducing the cost of human resources	Increasing the workload/reducing in-service trainings/applying temporary employment contracts	England and Spain [24, 59, 71, 77]		*	*							*	*		
Increasing the quality and flexibility of human resources	Training new healthcare professionals as an alternative to their migration/exporting healthcare professionals and training foreign students	Cuba [84, 89, 91]		*	*									*	
	Increasing healthcare personnel’s flexibility to reduce costs/	Brazil [93, 97]			*									*	
	Training in health promotion and epidemiology concepts for hospital professionals	Cuba [91]			*						*			*	
	Training primary healthcare professionals	Argentina [39]			*						*			*	

### Service delivery

Generally, countries with austerity policies (Brazil and Spain) adopted measures to promote the delivery of services by the private sector [58, 97, 98]. Most countries reduced hospital services and increased community and long-term services [24, 50, 58, 59, 77]. Countries with reform policies improved their primary care services [46, 50, 77, 84]. Coordination and integration between various health system levels was another strategy for cost reduction in Spain[58], England [50], Argentina [39] and Brazil [94]. Table 9 presents service delivery policies to counteract the economic crisis and their contribution in other dimensions of health system resilience.

**Table 9:** Service delivery policies to counteract economic crisis and their relationship to health system resilience components

Theme	Policies	Country	Resilience phases					Resilience attributes					
			Anticipation	Preparation	Response	Recovery	Growth	Awareness	Surge capacity	Flexibility	Resistance	Access to resources	Collaboration and coordination
Promotion of PHC	Development of family physicians and synchronization of the medical education system with it	Cuba [48, 91]		*			*		*	*			*
	Promoting PHC services	Brazil [87] and Argentina [39]		*			*		*	*			
	Integrating health service delivery systems based on PHC and the referral system	Cuba [76, 89, 91] Brazil [94] and England [50, 67]		*			*		*				*
	Increasing health promotion facilities including long-term care facilities	Cuba [46]		*	*	*	*		*	*		*	
	Closing continuing care centres	Spain [59]			*	*					*	*	
	Eliminating emergency care at the first level	Spain [58]			*	*					*	*	
Reducing hospital services	Creating low-cost hospital services	Cuba [46]		*			*		*		*		
	Creating new care models to provide more integrated care to reduce reliance on hospital services	England [50, 67]		*			*		*	*	*		*
	Reducing the pressure on hospitals through the implementation of the national program for the promotion of emergency care at the first level	Cuba [84]		*			*	*	*	*	*		*
	Increasing and promoting outpatient and emergency services	Cuba [84]		*			*		*	*		*	*
	Increasing waiting time	Brazil [74] Spain [58]			*	*					*	*	
	Closing hospital departments	Spain [24, 59]			*	*					*	*	
	Applying innovative hospital services management	Spain [59]		*			*		*	*			
	Development and encouragement of medium- and long-term care facilities to reduce length of stay in acute-care hospitals	Spain [58] England [50, 77]		*			*		*	*		*	*

Theme	Policies	Country	Resilience tools										Resilience strategies		
			Risk analysis	Change in input/output level	Change in quality level	Legislation	Change in behaviour	Planning	Monitoring	Institutionalization	Learning	Information and communication systems	Absorptive	Adaptive	Transformative
Promotion of PHC	Development of family physicians and synchronization of the medical education system with it	Cuba[48, 91]		*				*						*	
	Promoting PHC services	Brazil [87] and Argentina [39]		*						*				*	
	Integrating health service delivery systems based on PHC and the referral system	Cuba [76, 89, 91] Brazil [94] and England [50, 67]					*			*					*
	Increasing health promotion facilities including long-term care facilities	Cuba [46]		*						*				*	
	Closing continuing care centres	Spain [59]		*									*		
	Eliminating emergency care at first level	Spain [58]		*									*		
Reducing hospital services	Creating low-cost hospital services	Cuba [46]		*										*	
	Creating new care models to provide more integrated care to reduce reliance on hospital services	England [50, 67]								*	*				*
	Reducing the pressure on hospitals through the implementation of the national program for the promotion of emergency care at the first level	Cuba [84]	*		*		*		*	*				*	
	Increasing and promoting outpatient and emergency services	Cuba [84]			*					*				*	
	Increasing waiting time	Brazil [74] Spain [58]			*								*		
	Closing hospital departments	Spain [24, 59]		*									*		
	Applying innovative hospital services management	Spain [59]			*				*	*				*	
	Development and encouragement of medium- and long-term care facilities to reduce length of stay in acute-care hospitals	Spain [58] England [50, 77]					*			*	*			*	

## Resilience phases

Following the study, the contribution of various policies detected in each health system building block to resilience phases is discussed. For this purpose, the analysis of Tables 5, 6, 7, 8, and 9 was utilized.

### Anticipation phase

Anticipation phase policies are related to the building block of information systems, which can provide the necessary infrastructure for evidence-based policy-making in all other health system building blocks. Few adopted policies on anticipation focus on monitoring diseases and their related risk factors. For example, Spain and Brazil have implemented monitoring and assessment systems to evaluate population health risks [57, 59, 63, 87]. Additionally, Cuba implemented anticipatory policies aimed at reducing hospital expenses by improving emergency services and ensuring prompt patient admission [84].

### Preparation, response, recovery and growth phases

The analysis of the contribution of detected policies in resilience phases (Tables 5, 6, 7, 8, and 9) showed that nearly all growth-phase policies also contribute to the preparation of the health system for future crises. For example, policies adapted to promote PHC services will also provide the necessary infrastructure to deliver low-cost essential services during future crises [50, 67, 76, 87, 89, 91]. Additionally, policies adapted to recover the health system from an economic crisis are also a type of response-phase policy. Therefore, we analysed the response and recovery phases, as well as the growth and preparation phases, together and in relation to each other.

The analysis also showed that the governance policies are primarily focused on preparation and growth, as they adopted or transformed rules and regulation, or determined necessary roles and responsibilities by decisions regarding centralization or decentralization[39, 53, 58, 95] or decisions regarding PPP or privatization [24, 39, 50, 89, 93], while response and recovery policies are implemented in other health system building blocks such as financing, drugs and medical equipment, human resources and service delivery.

#### Response and recovery phases

Countries with austerity policies focused on the response and recovery phases by adopting measures to increasing privatization [24, 93]. This was achieved by reducing both the quality and quantity of financial and human resources [24, 59, 71, 77, 98, 100].

However, response and recovery policies in Cuba increased health system resources by enhancing health diplomacy and facilitating commercial relations with foreign institutions [89, 91]. Moreover, Cuba’s policies are aimed at improving the quality and quantity of medical equipment [89]. Additionally, in Germany, response and recovery policies increased the financial resources of health insurance funds, including increased contributions from patients, increases in taxes and increases in government assistance [52, 54, 96].

#### Growth and preparation phases

Cuba, Brazil, Argentina and England have prepared their health systems to counteract economic crises by enhancing low-cost service delivery infrastructures, which include expanding primary-care services [39, 50, 77, 84, 87]. Moreover, they have implemented cost control mechanisms in currently expensive service delivery infrastructures. These mechanisms include the implementation of clinical guidelines and protocols in England [77], as well as service rationing in Spain, Argentina and Brazil [24, 56, 59, 78, 79, 97, 99]. Concurrently, Brazil and Argentina have improved support for vulnerable groups and their health insurance [42, 79].

## Resilience attributes

The results of the policy analysis regarding their contribution to resilience attributes (Tables 5, 6, 7, 8, and 9) showed that there are limited awareness-raising policies in all countries. These policies aim to determine the cost–effectiveness of resource allocation. In Germany, Brazil, Spain and England, awareness policies have been implemented to identify the benefits of covered drugs and services [52, 63, 77, 87], with the aim of determining resource allocation. Additionally, monitoring of the financial capability of patients has been implemented to determine their insurance premium and services payments [24, 54, 56, 59].

Moreover, all countries aimed to improve resistance and access to resources during the crisis. However, in countries implementing reform policies, a higher percentage of these policies have simultaneously improved surge capacity, flexibility and collaboration and coordination. Conversely, in countries with austerity policies, the majority of resources, access and resistance policies were palliative, short-term measures.

Surge capacity is improved by enhancing the quality and efficiency of system inputs and processes, as well as by implementing flexible policies. Certain policies have improved collaboration and coordination among service providers (Cuba, Argentina, Spain and England) [50, 67, 76, 89, 91, 94] and health insurance funds (Germany and Argentina) [52, 54, 79, 96] and between countries for the import of health products (Cuba) [89, 91].

## Resilience tools

The analysis of various tools for resilient system application in health policies during economic crises led to the identification and introduction of four additional tools, including the following.

### Change in input and output levels

Such policies reduce or increase the health system’s inputs and outputs without any change in their structure or quality.

### Change in quality level

Such policies implicitly or explicitly intend to reduce costs by increasing or decreasing quality.

### Legislation

Such policies employ legal coercion.

### Behaviour

Such policies use incentives and penalties to change the behaviour of consumers or service providers to reduce costs.

The use of “Changes in input or output levels” tools has two main aspects. First, on one hand, countries expanded primary care (Cuba and England) [48, 50, 67, 84], reduced hospital services (England) [50, 67], increased support for vulnerable groups (Argentina) [39, 75, 79] and improved insurance funds (Germany and Argentina) [52, 54, 79, 96]. However, on the other hand, some countries have reduced governmental support and increased private sector contributions (Spain and Brazil) [24, 93] and decreased the number and salaries of healthcare personnel (England and Spain) [24, 77, 100].

The “change in quality level” tool was specifically used in four areas: primary care, hospital services, human resources and drugs and medical equipment. By applying this tool, Cuba, England and Argentina improved their family medicine, primary care management and human resources and the monitoring of health indicators such as maternal and child mortality rates [39, 50, 67, 76, 89, 91]. Also, Cuba increased the quality of hospital services by implementing “Hospital Home Program”, renewing medical equipment and enhancing access to cost–effective services [76, 89, 91]. Conversely, Brazil explicitly reduced quality improvement arrangements and eliminated quality control in private hospitals [93]. Additionally, the UK, Spain and Brazil reduced the quality of human resources through implicit policies of reducing the number of employees, increasing workloads and implementing inappropriate work contracts [58, 59, 77, 96].

The legislation tool was used to regulate the export and import of health products in Cuba [46], as well as their consumption in Spain, Brazil and England [24, 56, 77]. It also shaped the cost control behaviour of health service providers in Spain [24, 56] and enhanced health insurance funds in Argentina and Germany [52, 54, 79, 96].

Applying “behavioural change” tools for cost control involves developing appropriate service consumption through training and encouragement [50, 77, 84]. Conversely, resource consumption was controlled through regulatory and legislative mechanisms, evidence-based decision-making and the restructuring of purchaser–service provider relationships.

## Resilience strategies

**Fig. 3:**
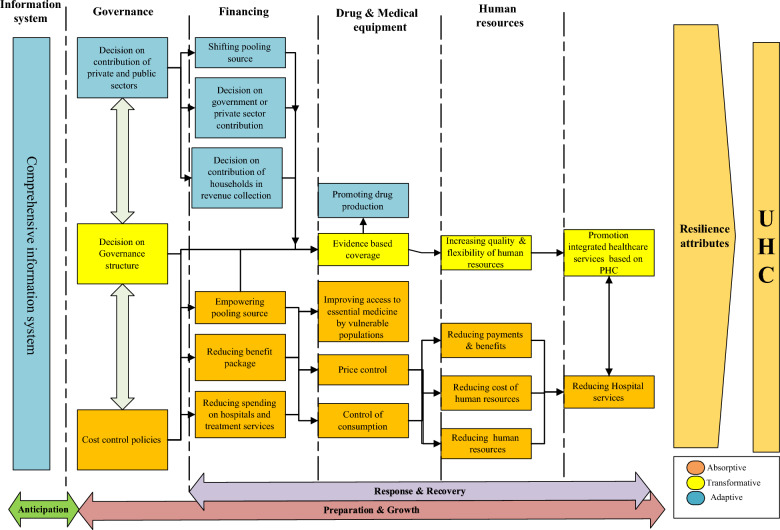
Adjusted resilience analysis meta-framework in context of economic crisis

In Fig. 3, the various policies implemented in response to the economic crisis are differentiated by colour according to the type of strategy employed. As shown in Fig. 3, the majority of cost control policies are absorptive policies. Accordingly, the successful implementation of these policies requires consideration of relevant adaptive and transformative policies in other health system building blocks.

## Discussion

Based on forecasts, currently, approximately 47% of low- and middle-income countries are adopting austerity policies as a result of budget cuts and rising debts [101]. Therefore, countries need a policy-making framework to enhance the resilience of their health systems during economic crises.

The analysis of various health systems’ experiences and their response to the economic crisis can provide an opportunity to identify the necessary contextual factors and strategies to achieve resilience in the health system [102]. This study aimed to adapt the resilience analysis meta-framework for policy-making, specifically in response to economic crises, using the best-fit framework synthesis method.

The adjusted framework (Fig. 3) illustrates the complementary and reciprocal relationships between resilience phases and health system building blocks (HSBB). Accordingly, failures in policy-making within each HSBB can lead to deficiencies in other HSBB implementations and ultimately hinder the achievement of a resilient health system. Indeed, resilience is the ability of complex adaptive systems (CAS). CAS constitute and are part of multiple interrelated subsystems. Hence, health system resilience policies should consider these subsystems and their relationships [103]. In other words, focusing on improving only one or two health system functions (such as service delivery) assumes that resilience is synonymous with performance improvement and treats the health system as an uncomplex entity [15].

Bozorgmehr et al., in their study, also raised the question of whether health system resilience is a feature or potential of health system to be achieved, or whether it is an outcome that can be measured. They referred to the RAMF, which considers resilience to be intermediate attributes guiding the health system to achieve its goals and improve its performance [104]. Additionally, the adjusted framework provides interrelated policy options, tools and strategies to achieve these attributes while considering the antecedents and the consequent of policies in other building blocks. The WHO, in its published toolkits on health system resilience, emphasized an integrated, whole-system approach to health system resilience [105]. However, in another report, the WHO considered resilience to be health system performance by providing various indicators in the building blocks of the health system and its goals which should be mapped, selected, targeted and measured after establishing measurement capacity. The results can be used to improve health system resilience [106]. Both studies provide a process: the adjusted RAMF outlines the process of improving resilience attributes to achieve universal health coverage, whereas the WHO study outlines the process of improving health system resilience measurement. The WHO also provides a roadmap to achieve health system resilience and refers to building health system resilience as a continual process requiring proactive and interrelated actions of various health sector and other relevant actors. It also considers resilience to be a prerequisite for achieving Universal Health Coverage (UHC) [107]. In the following, we explain the relationship between resilience phases and the health system building blocks in adjusted RAMF.

The main characteristic of a resilient health system is a dynamic information system with the ability to communicate between its various functions, subsystems or actors and maintain a robust surveillance system [15, 16]. The health system utilizes various tools, such as information and communication systems, monitoring and risk analysis to promote awareness, communication and coordination among different stakeholders during the anticipation phase.

The revised framework also emphasizes the priority of governance over other HSBBs. Indeed, governance is a characteristic of social systems and serves as the initial step in achieving health system resilience. It guides activities and communication networks between the other health system building blocks [15, 103, 108, 109]. Cuba, Argentina and England implemented institutionalization, coordination and collaboration tools to establish an integrated healthcare system, focusing on stewardship policies. The experience of coronavirus disease 2019 (COVID-19) also indicated the effects of coordination and collaboration among various system levels and institutions in achieving health system resilience [110]. As a solution to increase collaboration and coordination in response to the economic crisis and decrease competition, some countries have terminated public–private partnerships. Neoliberal policies are often cited as the cause of financial crises, as they undermine the responsibility of governments [70]. Additionally, a systematic review conducted in developing countries highlighted the failure of neoliberal policies – hospital autonomy reforms – in improving efficiency, accountability, quality and cost reduction [111]. Therefore, countries should exercise caution when selecting their approach to PPPs, especially in times of economic crisis.

An aware and integrated health system is capable of adopting evidence-based policies for reducing costs and allocating resources, including finances, equipment and human resources. Contrarily, some countries adopted short-term absorptive policies, including austerity measures, to combat the economic crisis. Defining health system priorities and identifying vulnerable areas will enable the restructuring of the healthcare system towards more integrated, cost–effective services. This will facilitate the achievement of the preparation and growth phases. This finding is consistent with that of Abimbola et al., who stated that adaptive and transformative strategies that contribute to health system preparedness and growth should address the deficiencies in the health system during the response and recovery phases. This includes addressing priority areas and identified needs. Otherwise, applying health system adaptation and transformation is referred to as coping and does not constitute resilience [112].

Studies have shown that these non-evidence-based cost-reduction policies jeopardize equity, access and the quality of health services [20, 113]. Indeed, several studies have revealed the detrimental effects of austerity policies. These effects include the reduction of human and financial resources, the limitation of service coverage and the hindrance of access to services. Austerity policies also lead to increased catastrophic costs and place additional pressure on vulnerable groups [72, 101, 113, 114]. Studies showed that these policies have been linked to higher mortality rates [64, 115] and decreasing life expectancy [116]. Thomson et al. propose increasing resource mobilization both internally and with the support of international organizations [113]. However, Stubbs et al. referred to the instability of certain internal financing mechanisms, such as donors, and the additional burden of debts on health systems [101]. This might relate to a failure in evidence-based decision-making [114]. Hence, these studies suggest the application and improvement of Health Technology Assessments (HTAs) to address stakeholders’ needs, prioritize resource allocation and enhance service delivery [72, 114].

Therefore, failure in evidence-based resource allocation policies can also jeopardize the goals of the health system. Linking insurance premiums and service copayments to people’s income quartile is an example of evidence-based resource allocation.

One of the primary limitations of this study is its reliance solely on a literature review. While literature reviews are valuable for theoretical insights, they are limited in their ability to validate practical effectiveness. This limitation may affect the applicability and generalizability of the findings to real-world settings. To strengthen the reliability and practical relevance of the findings, future research should incorporate a triangulation of methodologies. This can include empirical validation through qualitative and quantitative studies, such as case studies, surveys and interviews with key stakeholders in health systems.

## Conclusion

This study aimed to adjust the resilience analysis meta-framework for health system policy-making in response to economic crises using the best-fit framework synthesis method and a comparative analysis of countries’ experiences. While emphasizing the priority of the anticipation phase over other phases of creating resilience in the health system, the adopted RAMF highlights the absence of specific boundaries in the implementation of different resilience phases.

The revised framework demonstrates the interconnected and complementary relationships between resilience phases and health system building blocks. A resilient health system in the face of economic crises is integrated and aware, adopting evidence-based cost-reduction and resource allocation policies across all health system building blocks and resilience phases. This involves tools such as collaboration, coordination, institutionalization, legislation, behavioural changes, quality and quantity level changes and learning. The framework promotes integration and collaboration among various health system functions and actors, which is crucial for managing complex and uncertain situations. This framework can be further evaluated and refined in various contexts and settings to assess its feasibility and usefulness.

This best-fit framework synthesis provides practical examples for each RAMF dimension in the context of an economic crisis. It also presents general principles of resilience analysis that can be generalized to other crises and contexts.

The adjusted framework emphasizes the importance of an aware and integrated governance structure for appropriate decision-making regarding the role of the private sector in financing and service delivery, as well as the orientation of cost control policies. An aware and integrated health system governance makes decisions on the basis of needs and priorities. It shapes financing mechanisms to reduce the participation of vulnerable populations and allocates resources to health system priorities in the financing function. It also provides necessary and prioritized resources, including drugs, medical equipment and human resources, to cover the essential, low-cost and effective primary healthcare services. Such a system can focus on reducing high-cost hospital services on the basis of needs and priorities while expanding PHC. Conversely, the promotion of PHC services without an integrated and aware governance structure, which cannot shape appropriate evidence-based financing and resource creation, will face failure.

## Data Availability

No datasets were generated or analysed during the current study.
